# The Effect of Surface Hydroxyls on the Humidity-Sensitive Properties of LiCl-Doped ZnSn(OH)_6_ Sphere-Based Sensors

**DOI:** 10.3390/nano12030467

**Published:** 2022-01-28

**Authors:** Zhenjiang Li, Min Zhang, Linyu Yang, Rong Wu, Zhaofeng Wu, Youquan Jiang, Lina Zhou, Yanan Liu

**Affiliations:** 1 Xinjiang Key Laboratory of Solid State Physics and Devices, Xinjiang University, Urumqi 830046, China; zhenjiangli1210@163.com (Z.L.); yanglinyu0222@sina.com (L.Y.); JYQ3924@163.com (Y.J.); zln1761813466@163.com (L.Z.); LYN010601@163.com (Y.L.); 2 School of Physics Science and Technology, Xinjiang University, Urumqi 830046, China

**Keywords:** LiCl/ZnSn(OH)_6_, humidity sensor, surface hydroxyl, enhanced sensing performance

## Abstract

Pure zinc hydroxystannate (ZnSn(OH)_6_) and LiCl-doped ZnSn(OH)_6_ have been synthesized through a facile wet chemical method. The LiCl-doped samples keep their original spherical morphology as pure ZnSn(OH)_6,_ with some LiCl particles stuck to its surface, providing more active sites for the adsorption and desorption of water molecules. The influence of LiCl doping on the humidity-sensing properties was explored by varying the dopant concentration. The 16 wt% LiCl/ZnSn(OH)_6_ showed a better humidity-sensing performance than that of the pure ZnSn(OH)_6_ and other doped samples, including a high resistive sensitivity, a relatively small hysteresis, and a fast response speed. Through the FTIR analysis, the number of hydroxyl groups on the surface structure after aging has been found to decline markedly. These hydroxyl groups provide a platform for the adsorption of water molecules on the surface and promote the dissociation of water molecules. The detriment of aging to sensor performance should not be underrated. The complex impedance spectrum explains the mechanism of the sensor. These results demonstrate that ZnSn(OH)_6_ has potential application in fabricating humidity sensors, and the sensing performance of the sensor is enhanced by the dopant LiCl.

## 1. Introduction

In recent years, sensors have played an essential role in technological development of human society as the devices to record, transmit, process, and store information [1]. As one of the crucial branches of sensors, humidity sensors have been rapidly developed and are generally used in many application fields, including industrial process control, environment monitoring, corrosion estimation in industry, moisture monitoring in semiconductors, manufacturing and quality preservation of food [2,3], etc. The development of humidity sensors with shorter response and recovery time, higher sensitivity, wider humidity detection range, repeatability, long-term stability, and better linear response is the frontier field of sensor research. [4,5,6]. Hence, considerable attention is paid to looking for various sensing materials for humidity detection, such as metal oxide, ceramics, polymers, biomaterials, and 2D materials. [7,8,9,10,11,12]. The perovskite structure was formed from many oxides with transport properties ranging from predominantly ionic conduction to predominantly electronic conduction. Perovskite oxides can improve the reliability and long-term stability or performance because they could provide microstructural and morphological stability. In addition, the perovskite structure has two differently sized cations, which makes it amenable to a variety of dopant additions. This doping flexibility allows for control of the transport and catalytic properties to optimize sensor performance for particular applications [13,14,15,16].

ZnSn(OH)_6_, (ZHS) is a typical perovskite-structured hydroxide material [17]. In this compound, the metal atoms are octahedrally coordinated to oxygen atoms to form Sn(OH)_6_ and Zn(OH)_6_ polyhedra, which share their “O” corners to build the structural framework [18]. At the same time, ZHS is nontoxic, easy to prepare, and convenient to use, making it widely used in photocatalysts [19,20,21], gas sensors [22], flame retardants, smoke suppressants [23], lithium-ion batteries [24,25], biological monitoring [26], etc. It is well known that the microstructure, surface defects, and hydrophilic functional groups in the sensitive materials play an essential role in the performance of humidity sensors [27,28]. The main ZHS lattice contains a large number of hydroxide radicals. These hydroxide radicals are of great significance to the adsorption and desorption of water molecules. The sensitive mechanism of humidity sensors is known to be that their electrical characteristics (resistance or capacitance) change with the moisture absorption. Analyzing the crystal structure of ZHS [29], it is found that its surface contains a large number of hydroxyl groups, which combine with the hydroxyl produced by the absorbed water molecules to facilitate the detection of moisture signals. Therefore, it can be reasonably speculated that ZHS crystal has higher sensitivity to humidity signals. In addition, the wide bandgap of ZHS (4.35 eV) [30] endows higher stability to signal detection, making it more fascinating in microelectronic and optoelectronic areas.

In order to make the ZHS sensing material fully utilized, one of the most effective ways is to fabricate materials by combining ZHS with other humidity-sensing material dopants. The precious metals gold and silver are often selected as dopants and have been successfully doped into ZHS [19,31]. We have studied lithium chloride (LiCl). LiCl, as a strong electrolyte [32], is an attractive choice for constructing humidity-sensitive materials. With the increase in relative humidity and moisture adsorption by LiCl, the number of conductive ions produced by LiCl also increases, leading to a low resistivity and a decreased resistance [7,33,34]. In addition, LiCl shows an advantage of long-term working stability, so the humidity sensor based on LiCl has high precision and quick response characteristics [35].

The types of electrodes we commonly use are Dunmore, “sandwich”, interdigital electrode, and conventional sintered electrodes. Interdigital electrodes have the advantages of a wide humidity measurement range, high sensitivity, short response time, simple preparation process, good stability, and easy mass production [36,37,38,39].

However, as per our knowledge, the humidity properties of ZHS have been seldom involved. Here, ZHS nanocrystalline was synthesized as a sensing material. The humidity sensor is fabricated by coating this material on Ag-Pd interdigitated electrodes. Humidity-sensing research into impedance sensitivity and response speed was carried out. The complex impedance and infrared spectrum characteristics were recorded to explain the sensing mechanism and the effect of surface hydroxyl variation on humidity-sensing properties.

## 2. Materials and Methods

### 2.1. Materials

Zinc sulfate heptahydrate (ZnSO_4_·7H_2_O), potassium stannate trihydrate (K_2_SnO_3_·3H_2_O), ammonium hydroxide (NH_3_·H_2_O), lithium chloride (LiCl), magnesium chloride (MgCl), potassium carbonate (K_2_CO_3_), magnesium nitrate (Mg(NO_3_)_2_), copper chloride (CuCl_2_), sodium chloride (NaCl), potassium chloride (KCl), potassium nitrate (KNO_3_), formaldehyde (HCHO), ethanol (C_2_H_6_O), acetone (C_3_H_6_O), and hydrogen peroxide (H_2_O_2_) were purchased from Sinopharm Chemical Reagent Co., Ltd., Beijing, China. All the reagents used in this experiment were of analytical grade.

### 2.2. Synthesis of ZHS Precursor

The ZHS precursor was synthesized by a wet chemical method. In the experiment, 2.8756 g ZnSO_4_ ·7H_2_O and 2.9895 g K_2_SnO_3_·3H_2_O were dissolved in 100 mL and 20 mL deionized water, respectively. Subsequently, 8 mL of ammonia water was added to the clear ZnSO_4_·7H_2_O solution. Then, the previous K_2_SnO_3_ aqueous solution was dropped into the ZnSO_4_ and ammonia mixed solution under stirring at 60 °C for 6 h. The product was collected after the centrifugation process with washed by deionized water and alcohol several times. Finally, a white sample was obtained by drying at 60 °C for 6 h.

### 2.3. Synthesis of LiCl/ZHS Samples

**Figure 1:**
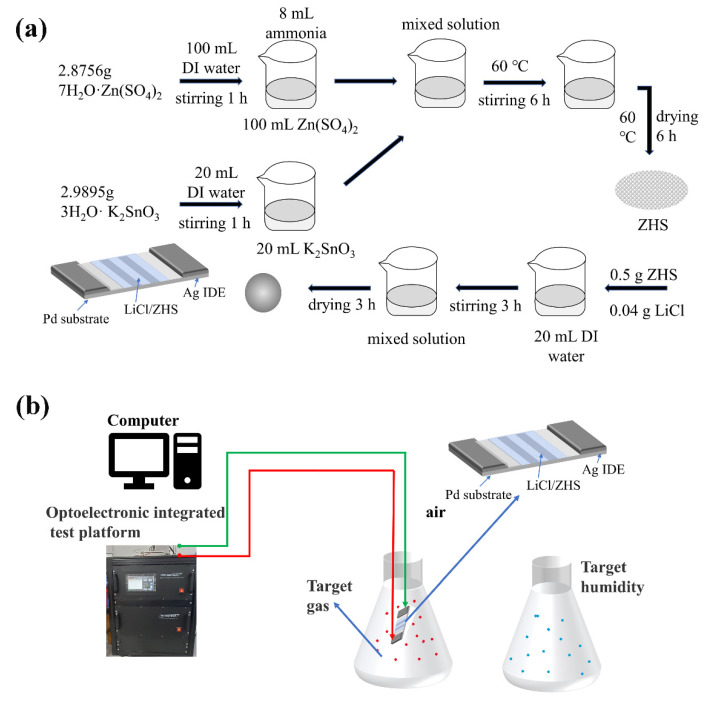
(**a**) Preparation of ZHS and LiCl/ZHS, (**b**) Characterization test diagram.

First, 0.5 g of ZHS sample was dissolved in 20 mL of deionized water to form a homogeneous solution under continuous stirring, into which different amounts (8 wt%, 12 wt%, 16 wt%, 20 wt%, and 24 wt%) of LiCl were added. After being aged for 3 h at room temperature, the resulting material were named 8 wt%, 12 wt%, 16 wt%, 20 wt%, and 24 wt% LiCl/ZHS. The synthesis process of ZHS and LiCl/ZHS is shown in Figure 1a.

### 2.4. Materials Characterization

The crystal structure of the samples was characterized by X-ray diffraction (XRD, Ultima IV, Rigaku, Tokyo, Japan) with Cu Kα radiation (λ = 1.5418 Å) at a scan rate of 4.5° min^−1^ and performed at an angle range of 15–80°. The specific surface areas of the products were measured and analyzed by means of a nitrogen adsorption–desorption instrument (BET) (ASAP 2460, Mike, Micromeritics Instrument Corporation, Norcross, GA, USA). The morphologies of all of the samples were characterized by a field emission scanning electron microscope (SEM, Zeiss sigma 500, Jena, Germany) at an accelerating voltage of 5 kV. The Fourier transform infrared spectroscopy (FT-IR) spectra of the materials were recorded with a Nicolet IS5 spectrometer (Thermo, Shanghai, China). A KBr pellet (Seebio, Shanghai, China) was used to prepare the samples for FTIR measurement the range of 500–4000 cm^−1^ and averaged 32 times.

### 2.5. Fabrication and Measurement of Humidity Sensors

To obtain the humidity sensors, the as-synthesized sample was mixed with deionized water in a weight ratio of 5:1 to form a paste. The paste was coated on a ceramic substrate (13 mm × 7 mm, 0.5 mm in thick) with five pairs of Ag-Pd interdigitated electrodes (electrodes width and distance: 0.15 mm) to form the sensitive film. The film was dried in air at 60 °C for 6 h. Finally, the humidity sensor was obtained. To test the humidity sensor characteristic, the ZAHNER-IM6EX electrochemical workstation (Zennium pro, Kronach, Germany) was used to measure the electrical properties of the sensors. The sensors were placed into eight chambers with different relative humidity at room temperature. The relative humidity of 11–95% RH was obtained using different saturated salt solutions as humidity sources. The saturated salt solutions were made from LiCl, MgCl_2_, K_2_CO_3_, Mg (NO_3_)_2_, CuCl_2_, NaCl, KCl, and KNO_3_ with the relative humidity of 11%, 33%, 43%, 54%, 64%, 75%, 85%, and 95%, respectively. In the humidity study, the voltage was set at 1 V AC, and the frequency changes from 40 Hz to 100 kHz. During the humidity sensitivity measurement, the prepared sensor was put into some chambers with different humidity, which were carried out by switching the sensor from one chamber quickly into another one. The sensors were aged in 100% RH chamber at 1 V AC and 100 Hz for about 3 h. All of the humidity-sensing measurements were carried out at 25 °C. The tests based on LiCl/ZHS sensors are similar to those reported previously [40]. The target vapor is generated by a thermal evaporation way according to Figure 1b. The anti-interference of the humidity sensor to HCHO, C_2_H_6_O, C_3_H_6_O, H_2_O_2,_ and NH_3_ five gases at a concentration of 500 ppm was tested at room temperature. The ambient humidity at room temperature is 30 RH%.

## 3. Results

**Figure 2:**
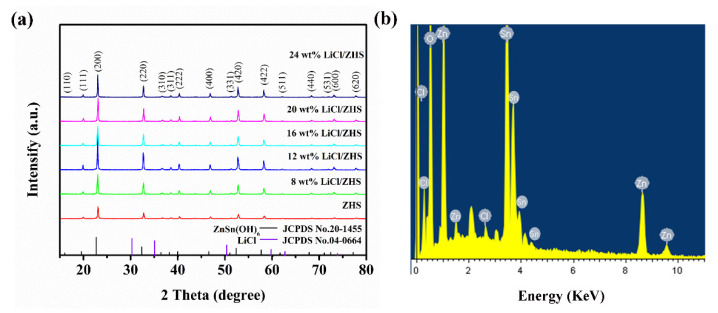
(**a**) The XRD patterns of pure ZHS, 8 wt%, 12 wt%, 16 wt%, 20 wt%, and 24 wt% LiCl/ZHS samples, (**b**) The corresponding energy-dispersive X-ray spectroscopy (EDS) spectra.

The crystal structure of pure and LiCl-doped ZHS samples was confirmed by the XRD analysis, as shown in Figure 2a. The structure of ZHS is cubic perovskite phase, which could be well confirmed by the diffraction patterns, consisting of the PDF card of ZHS JCPDS.No20-1455. The average crystallite sizes is 41.8 nm of ZnSn(OH)_6_, according to the Scherrer formula: D = kλ/βcos θ. When the amount of dopant is 0–24 wt%, the crystal size of LiCl/ZHS shows a downward trend. The full peaks of ZHS and LiCl/ZHS shift less than 1 degree to the high angle, indicating that the crystal plane spacing is decreasing. As for LiCl-doped ZHS samples, fraction peaks corresponding to the dopant were not found, indicating that the introduction of LiCl dopant does not change the original crystal structure. The strong diffraction peaks with no impurities indicate the good crystallinity and high purity of the samples. Figure 2b is the energy-dispersive spectrum analysis of the LiCl:ZHS sphere in the selected region of Figure 2b, which proved the existence of the Cl element (the element of Li cannot be detected owing to instrument reasons).

**Figure 3:**
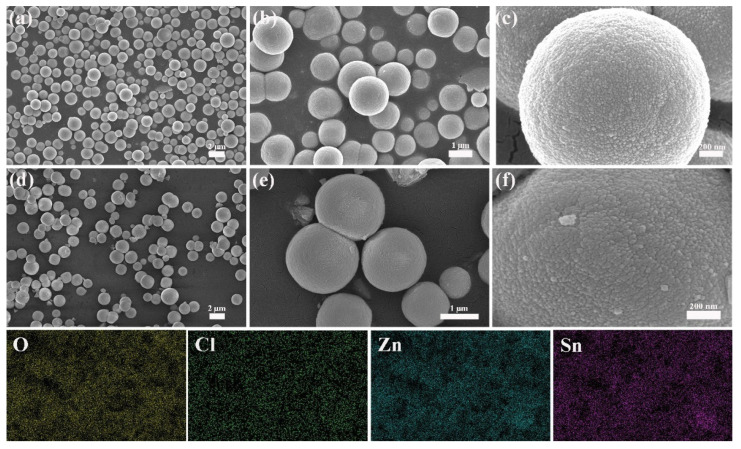
(**a**–**c**) The SEM of the pure ZHS under different magnification, (**d**–**f**) The SEM of the 16 wt% LiCl/ZHS samples under different magnification.

Figure 3 shows the morphology images of the pure ZHS and 16 wt% LiCl/ZHS samples at high and low magnification. As exhibited in Figure 3, the morphology of ZHS displays a sphere shape, which is formed by tiny nanoparticles with diameters of about 58 nm (as shown in Figure 3f). The SEM images of ZHS after the introduction of LiCl are shown in Figure 3d–f. It can be seen that the morphology of 16 wt% LiCl/ZHS is similar to that of ZHS, and the original pattern of spheres remains unchanged. It is worth noting that the surface of the spheres composed of small particles is not smooth, which makes it have a large surface area. The Brunauer–Emmett–Teller BET specific surface area for ZHS and 16 wt% LiCl/ZHS are 1.3 and 2.8 m^2^/g. From the element mapping analysis in Figure 3f, it can be observed that O, Cl, Zn, and Sn elements are evenly distributed in 16 wt% LiCl/ZHS. That also shows that the LiCl-doped ZHS is successfully prepared.

**Figure 4:**
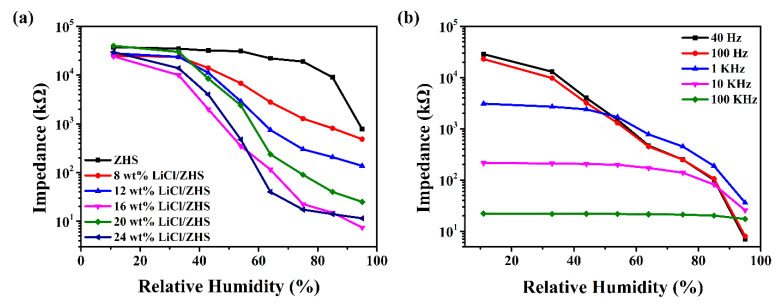
(**a**) The dependence of impedance on RH for pure and LiCl-doped ZHS spheres measured at 100 Hz and AC 1 V, (**b**) The dependence of impedance on RH for 16 wt% LiCl/ZHS sample measured at various frequencies.

Figure 4a presents the curves of the impedance variation with humidity for pure and LiCl-doped ZHS samples at 100 Hz, AC 1 V. The response of the pure ZHS-based sensor in the humidity that ranges from 11% to 95% RH is about two orders of magnitude. As a typical electrolyte material, LiCl has been widely used in humidity sensors [41]. The small radius of lithium ion produces a large polarization force, attracting water molecules rich in electrons in oxygen atoms. [42]. The performance of LiCl doped devices is very different from that of pure devices. The moisture sensitivity of all samples doped with lithium chloride is significantly improved, indicating that the addition of hydrophilic LiCl can greatly improve the water adsorption capacity. In addition, reversible water adsorption and desorption processes show that water molecules can easily diffuse in and out of the ZHS surface. Water adsorption and desorption are very important for humidity sensors because they determine the response speed of sensors [7]. The sensitivity of the 16 wt% LiCl/ZHS sample exhibits a better performance than that of other materials. A small amount of LiCl doping has little effect on the sensing performance of ZHS. When the amount of LiCl doping is large, the sensors show long response and recovery time, and the wet hysteresis difference is also increasing. Thus, the 16 wt% LiCl/ZHS sample was selected as the optimum doping sample for subsequent measurement.

Figure 4b exhibits the relationship between the impedance of 16 wt% LiCl/ZHS and RH at various frequencies (40 Hz, 100 Hz, 1 kHz, 10 kHz, 100 kHz). The operating frequency significantly influenced the humidity sensitivity performance. When the operating frequency is low, the impedance of the sensor produces a dramatic decrease. As the frequency increases further, the impedance tends to be flat with the RH variation. This result is due to the fact that the polarization process of water is lower than the change of electric field at high frequency [43]. Overall, the measurement at low frequency is unstable, and the sensitivity of the humidity sensor is low at high frequency. So, we chose 100 Hz as the best working frequency. When the operating frequency is set as 100 Hz, the impedance of the 16 wt% LiCl/ZHS humidity sensor drops from 23,000 to 8 kΩ as the humidity changes from 11% to 95% RH, contributing to a high sensitivity of about four orders of magnitude. Compared to the humidity sensor performance reported in previous work (Table 1), the LiCl/ZHS sensor had a high response and fast response/recovery speed.

**Figure 5:**
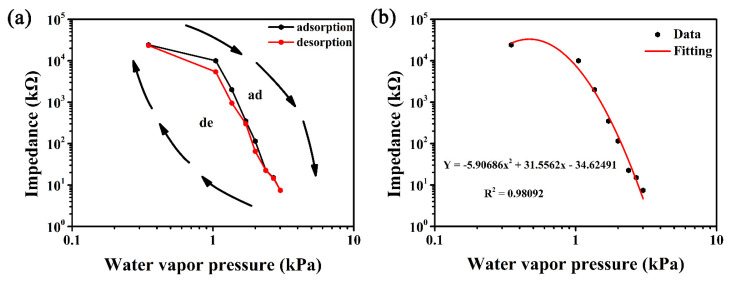
(**a**) The hysteresis characteristic curve for the 16 wt% LiCl/ZHS sample measured at 100 Hz and AC 1 V (**b**) The fitting curve between impedance and water vapor pressure of 16 wt% LiCl/ZHS sample.

Figure 5a demonstrates the humidity hysteresis characteristics of 16 wt% LiCl/ZHS based sensor within 11–95% RH, under the condition of 1 V AC voltage and 100 Hz. When our sensor devices test for different humidity levels, we do so manually by quickly switching between humidity environments, which may be affected by ambient air along the way. The adsorption and desorption of water molecules were highly reversible, and no obvious hysteresis was observed. In the range of 11–95% RH, the maximum wet hysteresis difference of the sensor is less than 9.5% RH. The fitting function of impedance Y and the water vapor pressure X of 16 wt% LiCl/ZHS humidity sensor is Y = −5.90686X^2^ + 31.5562X − 34.6291, and the regression coefficient R^2^ = 0.98092. Generally speaking, the lower the water vapor pressure, the higher the impedance of the sensing material. However, the maximum impedance can be seen on the fitted image at a water vapor pressure of 0.4 kPa, which may be caused by the statistical error of low relative humidity measurement not considered in the numerical fitting.

**Figure 6:**
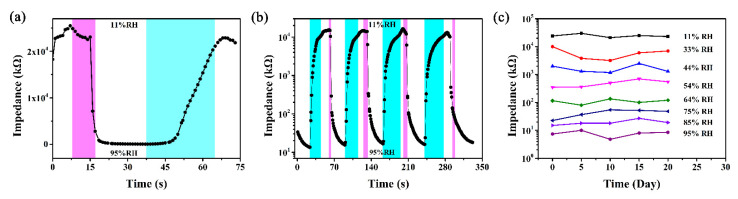
(**a**) The response and recovery characteristics curve of the 16 wt% LiCl/ZHS sensor between 11% and 95% RH, (**b**) The four periods response and recovery curve of the 16 wt% LiCl/ZHS sensor, (**c**) The stability test of the 16 wt% LiCl/ZHS humidity sensor in a range of 11% to 95% RH.

Response time refers to the time required for the sensor to reach 90% of the total impedance change during the moisture absorption and desorption. Figure 6 shows the response and recovery characteristics of the 16 wt% LiCl/ZHS-based humidity sensor after a single period and four periods. It is obvious from Figure 6a that the response time is less than 10 s, and the recovery time is shorter than 32 s. The four periods’ response and recovery characteristics exhibit the high stability and repeatability of the sensors reported herein. The average response and recovery time after four testing cycles were 11.35 s and 30.6 s, respectively. Stability is an important characteristic of sensors. The impedance changes of 16 wt% LiCl/ZHS humidity sensor from 11% to 95% RH were measured in 20 days. It can be observed that the impedance only fluctuates slightly under different RH, so this sensor can be used as a humidity measurement device.

**Figure 7:**
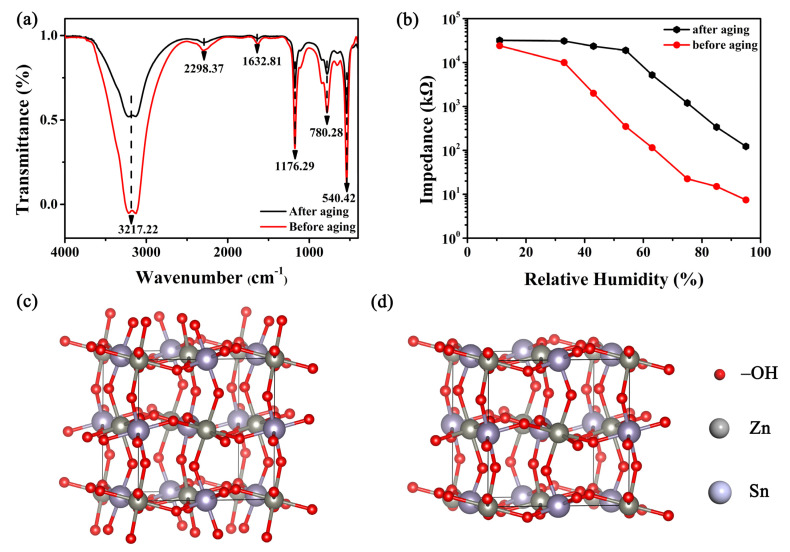
(**a**) The FTIR of 16 wt% LiCl/ZHS sample, (**b**) The humidity characteristic change of 16 wt% LiCl/ZHS before and after aging, (**c**) The crystal structure of ZHS before aging (**d**) and after aging.

The structure and functional groups are the key factors affecting the conductivity of sensing materials. In order to determine the functional groups on the film surface, Fourier infrared spectroscopy was performed, as displayed in Figure 7a. A broadband center around the range of 3000–3800 cm^−1^, especially a strong absorption peak at 3217 cm^−1^, was observed, which is attributed to the stretching of the hydroxyl groups [45]. In addition, a strong absorption peak caused by the Sn-OH bending vibration can be observed near 1176 cm^−1^ [45]. The absorption peak at 780 and 540 cm^−1^ is originated from the hydrogen bond between water molecules and the stretching modes of Sn-OH [46], respectively. The hydroxyls are known to be hydrophilic functional groups and possess high affinity for attracting water molecules. At the same time, these also help provide mobile protons to cause changes in the electrical properties and affect the proton transport mechanism in different humidity environments.

It is worth mentioning that the absorption peak at 3217 cm^−1^ shows distinct changes before and after aging. Compared with the decreased number of hydroxyl groups after aging, a large number of hydroxyl groups that exist before aging enable the samples to possess a strong adsorption capacity for water molecules. Such changes in the number of hydroxyls were also proved by analyzing the ZHS crystal structure and humidity characteristics before and after aging of the sensors, as shown in Figure 7b–d. The surface structure of ZHS was covered with hydroxyl functional groups before aging, the number of which dropped sharply after the aging process. It appears that the hydroxyl groups have been destroyed during the aging process. Consequently, the adsorption capacity of water molecules for the 16 wt% LiCl/ZHS sample was significantly reduced. The above analysis matches well with the humidity sensitivity characteristic curves exhibited in Figure 6b. Before aging, the 16 wt% LiCl sensors had high sensitivity and good linearity. The sensitivity of this sensor after aging reduces evidently, and the linearity becomes poor. This result is in good agreement with the outcome of FTIR analysis. Hence, it was inferred that the number of hydroxyl functional groups is crucial to the detection of humidity and performance of the humidity sensor. However, the absorption peak around 2298 cm^−1^ [47] is a result of the asymmetric stretching vibration of atmospheric CO_2_ or CO_2_ adsorbed on the ZHS sample. The absorption peak at 1632 cm^−1^ [48] is related to the in-plane vibration of the H-O-H bending band of the adsorbed water molecule or the C=C of the sp^2^ hybrid.

**Figure 8:**
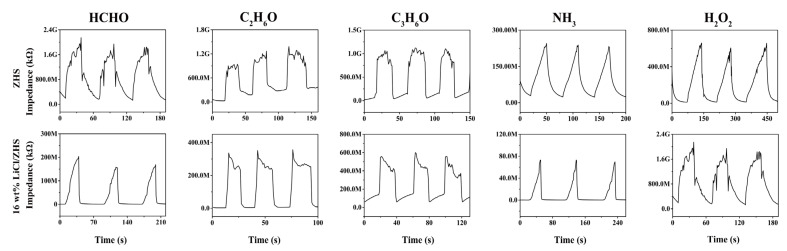
The response of the 16 wt% LiCl/ZHS sample to 500 ppm of HCHO, C_2_H_6_O, C_3_H_6_O, H_2_O_2_, and NH_3_.

To ensure the anti-interference of the ZHS and 16 wt% LiCl/ZHS humidity sensor in the presence of some other gas molecules in the air, we selected HCHO, C_2_H_6_O, C_3_H_6_O, H_2_O_2_, and NH_3_ at a concentration of 500 ppm to conduct the cross-sensitivity test, as shown in Figure 8. The response of the ZHS sensor to a concentration of 500 ppm of HCHO, C_2_H_6_O, C_3_H_6_O, and NH_3_ can be observed to be very small. Among all the gases, the sensor exhibits the highest response toward NH_3_. At the same time, the sensor has good repeatability and stability after different testing cycles. After introducing LiCl, the response of the 16 wt% LiCl/ZHS sensor to these gases improved dramatically, which is attributed to the active site induced by doping nanoparticles on the ZHS surface. Moreover, the response of the 16 wt% LiCl/ZHS-based sensor to NH_3_ (2.0 × 10^6^ Ω) is equivalent to that at 43% RH. Hence, the 16 wt% LiCl/ZHS humidity sensor before aging was immune to these gases in the 64–95% RH range. With regard to the highest response of ammonia, according to the widely accepted gas sensing theory, the sensitivity of the resistance gas sensor is related to the change of electron depletion layer. When the 16 wt% LiCl/ZHS sensor is exposed to the target gas, the surface will absorb oxygen (O_2_^−^, <100 °C; O^−^, 100–300 °C; O^2−^, >300 °C) with the following reaction equations:

O_2_ (gas) → O_2_ (ads) (1)

O_2_ (ads) + e^−^ → O_2_^−^ (ads) (2)

4NH_3_ (g) + 3O_2_^−^ (s) → 2N_2_ + 6H_2_O + 3e^−^. (3)

When the sensor is exposed to air, oxygen molecules capture electrons to form O_2_^−^, resulting in the formation of an electron depletion layer at the ZHS surface and the sample with a high resistance. When the sensor is placed in ammonia (NH_3_), O_2_^−^ would oxide ammonia molecules, and then, captured electrons are released back to the sample surface. The electron depletion layer narrows, the resistance decreases, and the current increases.

That means that the fabricated humidity sensor would work more stably in a high humidity environment.

**Figure 9:**
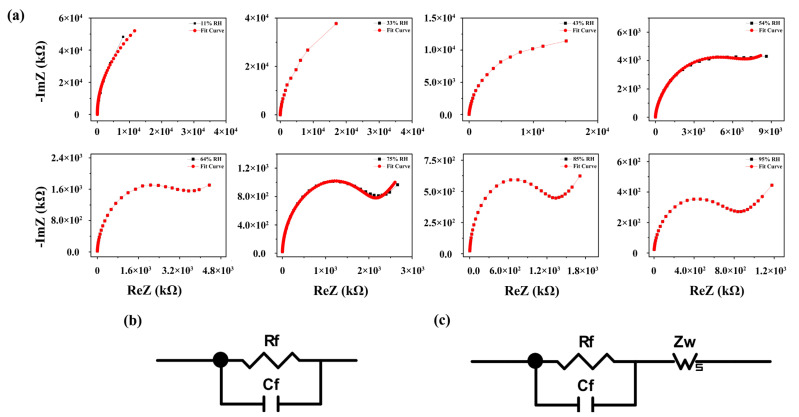
(**a**) The complex impedance spectroscopy (CIS) plots of 16 wt% LiCl/ZHS sensor at various RHs (**b**,**c**) and their corresponding equivalent circuits.

According to the above analysis of humidity-sensitive characteristics, the high sensitivity of the 16 wt% LiCl/ZHS humidity sensor mainly relies on the rich hydrophilic function groups on the material surface. Due to some hydrophilic functional groups (OH) on the surface of 16 wt% LiCl/ZHS, more water molecules can be adsorbed on the surface. Additionally, these water molecules can accelerate the dissociation ability of conductive ions, which has achieved the purpose of enhancing ion conduction. To better analyze the humidity-sensing mechanism, we tested the complex impedance spectrum (Cole–Cole diagram) of the 16 wt% LiCl/ZHS samples and fitted an equivalent circuit to study the electrical characteristics of the 16 wt% LiCl/ZHS sensor. The real part ReZ and the imaginary part ImZ of the device impedance were measured under different ambient humidity in the frequency range from 40 Hz to 100 kHz, as shown in Figure 9.

Based on the physical theory of electrolytes, we know that in the equivalent circuit (EC), the resistance represents the conducting process, the capacitance represents the polarization process, and the Warburg impedance (Z_w_) is the impedance caused by diffusion, which usually occurs in the low-frequency band. The CIS curve of 11% RH and 33% RH displays a straight line approximately parallel to the Y-axis. The impedance of sensors is very large. A few water molecules would be chemisorbed at a low humidity environment on the active sites of the 16 wt% LiCl/ZHS surface to form hydroxyl groups. Protons are transferred from these surface hydroxyls to water molecules to form H_3_O^+^. At this time, the proton works as the major carrier to realize hopping conduction. Around 43–54% RH, the impedance decreases further. The curve is semicircular. With the increase in adsorbed water molecules, the impedance decreases continuously, the protons and water molecules bind together to form H_3_O^+^. At the same time, some Li^+^ are ionized from the 16 wt% LiCl/ZHS sample. Hence, both H_3_O^+^ and Li^+^ participate in the conduction. Hence, at low RH levels (<54% RH), the EC can be regarded as a parallel connection of capacitor Cf and resistor Rf in this situation, as shown in Figure 9b [42]. At 64–85% RH, the CIS curve is displayed, which is composed of a semicircle and an obvious trailing line. This is due to the effect of electroactive material diffusion, resulting in a semicircular tail line assigned to Z_w_. As the humidity increases further, more and more water molecules are adsorbed onto the surface, tending to form a liquid layer. H_3_O^+^ releases a proton to a neighboring water molecule in the liquid water layer, which receives it while releasing another proton. Protons can move freely in a continuous layer of liquid water known as the Gaussian conduction mechanism (H_2_O + H_3_O^+^ → H_3_O^+^ + H_2_O) [49]. The Grotthuss chain reaction typically occurs at the low frequencies with the appearing of a trailing line in the complex impedance spectrum. Therefore, at high RH levels, the CIS curve gradually presents a straight line in the low-frequency region, and it keeps the semicircle unchanged in the high-frequency region. The equivalent circuit is accompanied by the appearance of Z_w_ in Figure 9c, which is reflected in the rising tail of the curve in the spectrum, as shown in Figure 9a [43,50].

LiCl would dissolve in the formed liquid water layer, and the dissociated Li^+^ and Cl^-^ also act as conduction carriers. At 95% RH, the CIS curve shows a semicircle and an upward tail. More and more water molecules are adsorbed at this time. The ion conduction of H_3_O^+^ and Li^+^ is further enhanced, resulting in a sharp reduction in the impedance. As a result, through the analysis of complex impedance spectrum at low and high humidity ambient, both proton and ionic conduction are found to exist in the humidity-sensing mechanism of the 16 wt% LiCl/ZHS sample.

## 4. Conclusions

In conclusion, the synthesis and characterization of pure ZHS and LiCl/ZHS samples were explored. After that, sensors based on ZHS with different doping content were fabricated, and their humidity-sensing properties were measured. As a typical electrolyte material, Li^+^ have a small radius and produce a large polarization force to attract electron-rich water molecules in oxygen atoms. The dopant LiCl dramatically improved the specific surface area of the pure ZHS sample and greatly enhanced the humidity-sensing response of the sensors. Among all the doping samples, the 16 wt% LiCl/ZHS exhibits a high sensitivity of about four orders of magnitude, the best linear correlation from 11–95% RH, a narrow humidity hysteresis of less than 9.5% RH, and a favorable response speed shorter than 10.2 s. According to Fourier infrared functional group analysis, the number of hydroxyl groups after aging is significantly reduced compared with that before aging. These hydroxyl groups contribute much to the adsorption of water molecules, which is highly consistent with the analysis of the ZHS crystal structure before and after aging. The complex impedance spectrum analysis reveals that the protons and introduced Li ions are the major conductive carriers. Protons and Li ions play a prominent role in low humidity and high environment, respectively. Both protons and Li ions work at a medium moisture ambience. These results demonstrate the superior performance of ZHS in effective humidity detection and the obvious improvement of humidity properties by LiCl doping.

## Figures and Tables

**Table 1:** The performance of the LiCl/ZHS humidity sensor compared to previously published work.

Material	Order of Impedance Change	Response Time (s)	Recovery Time (s)	Sensing Range (% RH)	Ref.
Er-ZnO	3	32.3	39.6	11–95	[44]
CdS/ZnO	-	110	32	11–95	[31]
LiCl/Pebax 2533	4	30	80	11–95	[32]
LiCl/PETMP-DVB	2	3.5	63	11–95	[7]
LiCl/ZHS	~4	<10	32	11–95	This work

## Data Availability

The data presented in this study are available within the present article.
